# Gestational diabetes mellitus, prenatal maternal depression, and risk for postpartum depression: an Environmental influences on Child Health Outcomes (ECHO) Study

**DOI:** 10.1186/s12884-022-05049-4

**Published:** 2022-10-08

**Authors:** Lauren C. Shuffrey, Maristella Lucchini, Santiago Morales, Ayesha Sania, Christine Hockett, Emily Barrett, Kecia N. Carroll, Camille C. Cioffi, Dana Dabelea, Sean Deoni, Anne L. Dunlop, Arielle Deutsch, William P. Fifer, Morgan R. Firestein, Monique M. Hedderson, Melanie Jacobson, Rachel S. Kelly, Jean M. Kerver, W. Alex Mason, Hooman Mirzakhani, Thomas G. O’Connor, Leonardo Trasande, Scott Weiss, Rosalind Wright, Yeyi Zhu, Rosa M. Crum, Seonjoo Lee, Amy J. Elliott, Catherine Monk, program collaborators for Environmental influences on Child Health Outcomes

**Affiliations:** 1 Department of Psychiatry, Division of Developmental Neuroscience, Columbia University Irving Medical Center, NYSPI, Pardes Rm 4932, 1051 Riverside Drive, New York, NY 10032 USA; 2 Department of Psychology, University of Southern California, Los Angeles, CA USA; 3 Avera Research Institute, Sioux Falls, SD USA; 4 Rutgers School of Public Health, Environmental and Occupational Health Sciences Institute, Piscataway, NJ USA; 5 Icahn School of Medicine at Mount Sinai, New York, NY USA; 6 Prevention Science Institute, University of Oregon, Eugene, OR USA; 7 Lifecourse Epidemiology of Adiposity and Diabetes (LEAD) Center, University of Colorado Anschutz Medical Campus, Aurora, CO USA; 8 Warren Alpert Medical School at Brown University, Providence, RI USA; 9 Department of Gynecology & Obstetrics, Emory University School of Medicine, Atlanta, GA USA; 10 Kaiser Permanente Northern California, Oakland, CA USA; 11 Department of Pediatrics, Division of Environmental Pediatrics, New York University School of Medicine, New York, NY USA; 12 Channing Division of Network Medicine, Brigham and Women’s Hospital, Boston, MA USA; 13 Department of Epidemiology and Biostatistics, Michigan State University, East Lansing, MI USA; 14 Department of Preventive Medicine, University of Tennessee Health Science Center, Memphis, TN USA; 15 Harvard Medical School, Boston, MA USA; 16 Department of Psychiatry, Psychology, Neuroscience, and Obstetrics and Gynecology, University of Rochester, Rochester, NY USA; 17 Department of Pediatrics, New York University Grossman School of Medicine, New York, NY USA; 18 Brigham and Women’s Hospital, Harvard Medical School, Boston, MA USA; 19 Kaiser Permanente Division of Research, Oakland, CA USA; 20 Johns Hopkins University, Baltimore, MD USA; 21 Mailman School of Public Health, Columbia University, New York, NY USA; 22 Department of Obstetrics and Gynecology, Columbia University Irving Medical Center, NY New York, USA; 23 Department of Psychiatry, Division of Behavioral Medicine, Columbia University Irving Medical Center, New York State Psychiatric Institute, New York, NY USA

**Keywords:** Gestational diabetes mellitus (GDM), Perinatal depression, Maternal mood disorders, Maternal metabolic disorders

## Abstract

**Background:**

Prior research has demonstrated bidirectional associations between gestational diabetes mellitus (GDM) and perinatal maternal depression. However, the association between GDM, prenatal depression, and postpartum depression (PPD) has not been examined in a prospective cohort longitudinally.

**Methods:**

Participants in the current analysis included 5,822 women from the National Institutes of Health’s Environmental influences on Child Health Outcomes (ECHO) Research Program: *N* = 4,606 with *Neither GDM nor Prenatal Maternal Depression (*Reference Category); *N* = 416 with *GDM only*; *N* = 689 with *Prenatal Maternal Depression only*; and *N* = 111 with *Comorbid GDM and Prenatal Maternal Depression*. The PROMIS-D scale was used to measure prenatal and postnatal maternal depressive symptoms. Primary analyses consisted of linear regression models to estimate the independent and joint effects of GDM and prenatal maternal depression on maternal postpartum depressive symptoms.

**Results:**

A higher proportion of women with GDM were classified as having prenatal depression (*N* = 111; 21%) compared to the proportion of women without GDM who were classified as having prenatal depression (*N* = 689; 13%), however this finding was not significant after adjustment for covariates. Women with *Comorbid GDM and Prenatal Maternal Depression* had significantly increased postpartum depressive symptoms measured by PROMIS-D T-scores compared to women with *Neither GDM nor Prenatal Maternal Depression* (mean difference 7.02, 95% CI 5.00, 9.05). *Comorbid GDM and Prenatal Maternal Depression* was associated with an increased likelihood of PPD (OR 7.38, 95% CI 4.05, 12.94). However, women with *GDM only* did not have increased postpartum PROMIS-D T-scores or increased rates of PPD.

**Conclusions:**

Our findings underscore the importance of universal depression screening during pregnancy and in the first postpartum year. Due to the joint association of GDM and prenatal maternal depression on risk of PPD, future studies should examine potential mechanisms underlying this relation.

**Supplementary Information:**

The online version contains supplementary material available at 10.1186/s12884-022-05049-4.

## Background

Gestational diabetes mellitus (GDM) is defined as diabetes diagnosed in the second or third trimester of pregnancy that was not clearly present prior to gestation [1]. GDM affects approximately 8.2% of pregnancies in the United States (US) [2] and approximately 14% of pregnancies globally with significant variability in prevalence based on diagnostic criteria, sociodemographic characteristics, and geographic region [3, 4]. GDM is associated with increased risk of birth complications and adverse long-term health outcomes for mothers including cardiometabolic conditions [5]. A growing body of research in pregnant and non-pregnant populations suggests a bidirectional association between diabetes and depression [6–10]. Prior studies have demonstrated women with depression prior to pregnancy are more likely to be diagnosed with GDM and women with GDM are 1.5 times more likely to be diagnosed with postpartum depression (PPD) [11–18]. PPD is among the most common perinatal morbidities affecting approximately 17% of women globally with adverse consequences for maternal health, well-being, and self-care [19, 20].

A recent meta-analysis (2020) examined the association between GDM and depressive symptoms around the time of GDM diagnosis, subsequent to GDM diagnosis, and in the postpartum period [21]. Despite significant heterogeneity in participant sociodemographic characteristics across included studies, GDM was associated with increased prenatal depressive symptoms around the time of GDM diagnosis with a pooled odds ratio (OR) of 2.08 (95% CI 1.42, 3.05) and increased prenatal depressive symptoms following GDM diagnosis with a pooled OR of 1.41 (95% CI 0.88, 2.25) [21]. GDM status also was associated with PPD with a pooled OR of 1.59 (95% CI 1.26, 2.00) [21]. Prior research from US based samples also indicates GDM and perinatal depression may both disproportionally affect women who self-identify as non-White or Hispanic [22–26], likely due to health determinants such as such as poverty, systemic racism, history of trauma, and limited access to health and mental health care resources [27, 28]. Specifically, studies examining racial or ethnic disparities in pregnancy conditions have reported women who identify as Hispanic or Latina and/or Asian have an increased incidence of GDM compared to non-Hispanic White women [23]. For example, approximately 15% of women who self-identify as Southeast Asian develop GDM during pregnancy compared to approximately 4% of non-Hispanic White women [22]. Studies examining the incidence of perinatal mood disorders have reported Black or African American and American Indian/Alaskan Native women have increased incidence of perinatal depression compared to non-Hispanic White women [24].

Despite increased risk of adverse long-term outcomes for both mothers and their children, the association between comorbid GDM and prenatal depression in relation to PPD risk has not been examined in a single longitudinal analysis using a prospective cohort design. Studies to date have assessed associations between GDM and prenatal or postnatal depression rather than examining their comorbidity in predicting PPD or postpartum symptoms. Thus, our objective was to determine the joint and independent associations of GDM and prenatal maternal depression on PPD. Given the high co-occurrence of these disorders, particularly in diverse populations, there is important public health and clinical value to understanding their impact on PPD with implications for both maternal and child well-being.

## Methods

### Study design and participants

**Fig. 1:**
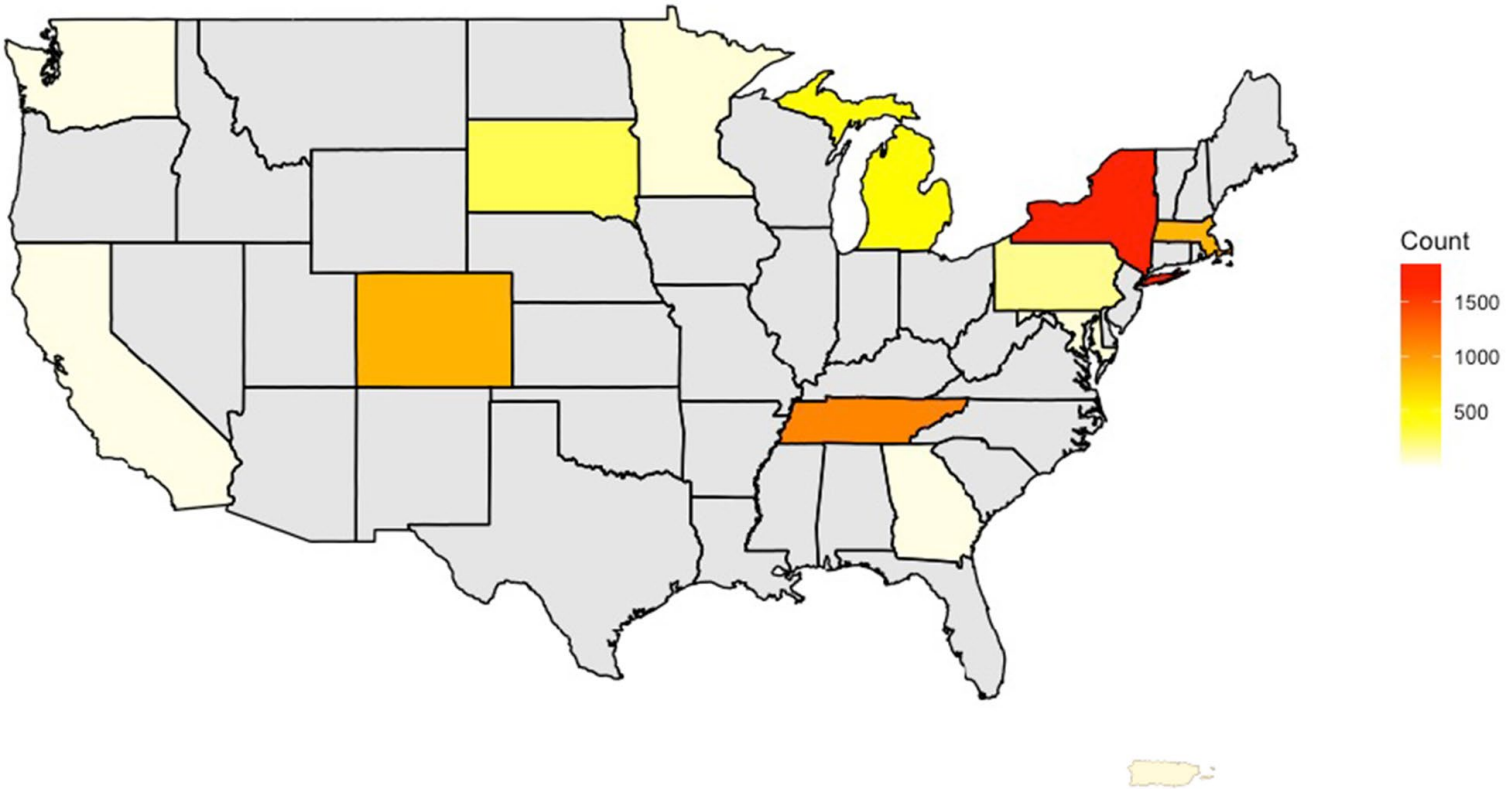
Distribution of Participants Across the United States and Puerto Rico. Distribution of 5,822 maternal participants from 16 ECHO cohorts enrolled from thirteen US states and Puerto Rico where the color bar represents the count per state. Figure 1 was generated in R version 4.1.3 using the ‘ggplot [29]’, ‘maps [30]’, and ‘mapproj [31]’ libraries

Data for this analysis were collected through the National Institutes of Health’s (NIH) Environmental influences on Child Health outcomes (ECHO) Research Program. Participants in the current analysis included pregnant individuals with complete information regarding GDM status (GDM during pregnancy vs. no GDM during pregnancy), at least one prenatal self-reported depression assessment, and a minimum of one self-reported postnatal depression assessment collected during the first postpartum year (*N* = 5866). Exclusion criteria consisted of Type 1 or Type 2 diabetes. After exclusions a total of 5,822 maternal participants across 16 cohorts, enrolled from thirteen US states and Puerto Rico, met criteria and were included in the current analysis (Fig. 1). The study protocol was approved by the local or single ECHO institutional review board. Written informed consent was obtained from all participants for ECHO-wide Cohort Data Collection Protocol participation and for participation in specific cohorts. Both a central and cohort-specific institutional review board monitored human subject activities at each cohort site and the centralized ECHO Data Analysis Center. The ECHO-wide protocol is approved under a single IRB, which is of Western Institutional Review Board (WIRB) Copernicus Group IRB. CG IRB is registered with the Office for Human Research Protections (OHRP and FDA) as IRB00000533.

### Gestational diabetes mellitus

Information regarding GDM during pregnancy was harmonized across ECHO cohorts (Supplement). All participants had complete information regarding GDM status during pregnancy.

### NIH Patient-Reported Outcomes Measurement Information System Depression Scale (PROMIS®-D)

The PROMIS-D scale [32] was utilized by the NIH’s ECHO Research Program to harmonize various different self-report depression instruments used by individual cohorts to one common scale (Supplement). PROMIS-D T-scores are referenced to a mean of 50 and standard deviation of 10 with respect to the general adult US population, such that individuals with T-scores of 50 have depressive symptom severity equal to the mean in the general adult US population. PROMIS-D cutoff scores were derived from item response theory (IRT)-based differential item function (DIF) analyses suggesting specific cutoffs based on the original assessment scale harmonized to PROMIS-D T-scores (see supplement). In the absence of IRT based cutoff scores, a PROMIS-D T-score cutoff of 60 or higher was utilized to indicate clinical levels of depression based on existing literature [33–35].

### Sociodemographic and medical history

Covariates described below were harmonized across ECHO cohorts based on either maternal self-report using ECHO case report forms or through maternal-infant medical record abstraction.

#### Statistical analyses

All statistical analyses were conducted in R version 4.1.0 in a secure virtual private network platform hosted by the Research Triangle Institute (RTI) using de-identified data. Demographic differences between the GDM and non-GDM groups were examined using Chi Square or Fisher’s Exact Test. We examined if women with GDM had increased prenatal depressive symptoms using linear regression models in unadjusted, partially adjusted, and fully adjusted models (described below). We additionally examined if women with GDM were more likely to be classified as having clinical levels of prenatal maternal depression based on PROMIS-D cutoff scores using Chi Square analysis. *Post-hoc* analyses analysis consisted of logistic regression to examine the likelihood of prenatal maternal depression based on PROMIS-D cutoff scores by GDM status in unadjusted, partially adjusted, and fully adjusted models (described below). Primary analyses consisted of linear regression models to estimate the independent and joint effects of GDM and prenatal maternal depression on maternal postpartum depressive symptoms operationalized by continuous PROMIS-D T-scores during the first postpartum year. We created a GDM-depression variable with four categories:

- (1) Neither GDM nor Prenatal Maternal Depression (*N* = 4,606, Reference Category);
- (2) GDM only (*N* = 416);
- (3) Prenatal Maternal Depression Only (*N* = 689).
- (4) Comorbid GDM and Prenatal Maternal Depression (*N* = 111).

GDM-depression groups were created on the basis of maternal GDM status (yes or no) and based on PROMIS-D cutoff scores to indicate clinical levels of depression (prenatal maternal depression yes or no). We implemented unadjusted, partially adjusted, and fully adjusted models. Unadjusted models included only the original depression instrument as a covariate. Partially adjusted models additionally adjusted for maternal race, ethnicity, age at delivery in years, highest educational attainment, gestational hypertension, and pre-pregnancy body mass index (BMI) categorized as either underweight/normal-weight or overweight/obese. Fully adjusted models additionally adjusted for delivery mode (vaginal vs. cesarean) and preterm delivery (less than 37 weeks’ gestation). Covariates included in partially and fully adjusted models were chosen a priori based on prior research in this domain. Missing data for covariates in the partially and fully adjusted models were handled using the missing-indicator method (Tables 1 and 2). We report standardized mean differences with confidence intervals (95% CI) and estimated marginal means for the fully adjusted models as our primary analysis. Secondary analyses consisted of logistic regression to examine the likelihood of maternal PPD based on PROMIS-D cutoff scores by GDM-depression category. *Post-hoc* analyses consisted of refitting primary fully adjusted models to estimate the independent and joint effects of GDM and prenatal maternal depression on maternal postpartum depressive symptoms stratified separately by Hispanic ethnicity and pre-pregnancy BMI categorized as overweight or obese (Supplement). In a sensitivity analysis, we refit a fully adjusted model including a cohort that contributed ≥ 20% of overall data (Supplement). The significance threshold for all analyses was set at *p* < 0.05.

**Table 1:** Maternal demographic information by GDM Status in ECHO participants

	**GDM** **(*N* = 527)**	**Non-GDM** **(*N* = 5295)**	**Overall** **(*N* = 5822)**
**Race**			
American Indian or Alaskan Native	< 5 (< 1%)	< 45 (< 1%)	< 50 (< 1%)
Asian	45 (8.5%)	197 (3.7%)	242 (4.2%)
Black	71 (13.5%)	1328 (25.1%)	1399 (24.0%)
Mixed Race	119 (22.6%)	636 (12.0%)	755 (13.0%)
Native Hawaiian or Other Pacific Islander	< 35 (< 5%)	< 35 (< 1%)	< 55 (< 1%)
White	259 (49.1%)	2993 (56.5%)	3252 (55.9%)
Missing	< 10 (< 2%)	< 70 (< 2%)	< 80 (< 2%)
**Ethnicity**			
Hispanic or Latino	210 (39.8%)	1015 (19.2%)	1225 (21.0%)
Non-Hispanic or Latino	315 (59.8%)	4273 (80.7%)	4588 (78.8%)
Missing	< 5 (< 1%)	< 10 (< 1%)	< 10 (< 1%)
**Highest Educational Attainment**			
Less than high school	63 (12.0%)	328 (6.2%)	391 (6.7%)
High School degree, GED, or equivalent	89 (16.9%)	565 (10.7%)	654 (11.2%)
Associates degree or trade school	95 (18.0%)	802 (15.1%)	897 (15.4%)
Bachelor's degree	83 (15.7%)	1070 (20.2%)	1153 (19.8%)
Master's degree or higher	111 (21.1%)	1146 (21.6%)	1257 (21.6%)
Missing	86 (16.3%)	1384 (26.1%)	1470 (25.2%)
**Age at Delivery (years)**			
Mean (SD)	32.5 (6.21)	30.0 (5.94)	30.2 (6.00)
Missing	< 5 (< 1%)	< 5 (< 1%)	< 10 (< 1%)
**Pre-pregnancy BMI Category**			
Underweight or normal weight	152 (28.8%)	2563 (48.4%)	2715 (46.6%)
Overweight or obese	318 (60.3%)	2419 (45.7%)	2737 (47.0%)
Missing	57 (10.8%)	313 (5.9%)	370 (6.4%)
**Delivery Mode**			
Vaginal	320 (60.7%)	3703 (69.9%)	4023 (69.1%)
Cesarean	194 (36.8%)	1414 (26.7%)	1608 (27.6%)
Missing	13 (2.5%)	178 (3.4%)	191 (3.3%)
**Preterm Birth (< 37 weeks’ gestation)**			
Term	478 (90.7%)	4936 (93.2%)	5414 (93.0%)
Preterm	49 (9.3%)	359 (6.8%)	408 (7.0%)

**Table 2:** Maternal demographic information by GDM-Prenatal Depression Group

	**No GDM or Prenatal Maternal Depression** **(*N* = 4606)**	**GDM Only** **(*N* = 416)**	**Prenatal Maternal Depression Only** **(*N* = 689)**	**GDM and** **Prenatal Maternal Depression** **(*N* = 111)**	**Overall Sample** **(*N* = 5822)**
**Race**					
American Indian or Alaskan Native	40 (0.9%)	< 5 (< 1%)	< 5 (< 1%)	< 5 (< 1%)	< 50 (< 1%)
Asian	152 (3.3%)	34 (8.2%)	45 (6.5%)	11 (9.9%)	242 (4.2%)
Black	1187 (25.8%)	59 (14.2%)	141 (20.5%)	12 (10.8%)	1399 (24.0%)
Mixed Race	470 (10.2%)	88 (21.2%)	166 (24.1%)	31 (27.9%)	755 (13.0%)
Native Hawaiian or Other Pacific Islander	< 35 (1%)	< 15 (< 4%)	< 10 (2%)	< 10 (6%)	< 55 (1%)
White	2680 (58.2%)	213 (51.2%)	313 (45.4%)	46 (41.4%)	3252 (55.9%)
Missing	< 55 (< 2%)	< 10 (< 2%)	< 14 (< 3%)	< 5 (4%)	< 80 (< 2%)
**Ethnicity**					
Hispanic or Latino	768 (16.7%)	152 (36.5%)	247 (35.8%)	58 (52.3%)	1225 (21.0%)
Non-Hispanic or Latino	3832 (83.2%)	264 (63.5%)	441 (64.0%)	51 (45.9%)	4588 (78.8%)
Missing	< 5 (< 1%)	< 5 (< 1%)	< 5 (< 1%)	< 5 (< 1%)	< 10 (< 1%)
**Highest Educational Attainment**					
Associates degree or trade school	673 (14.6%)	72 (17.3%)	129 (18.7%)	23 (20.7%)	897 (15.4%)
Bachelor's degree	933 (20.3%)	65 (15.6%)	137 (19.9%)	18 (16.2%)	1153 (19.8%)
High School degree, GED, or equivalent	452 (9.8%)	64 (15.4%)	113 (16.4%)	25 (22.5%)	654 (11.2%)
Less than high school	243 (5.3%)	48 (11.5%)	85 (12.3%)	15 (13.5%)	391 (6.7%)
Master's degree or higher	978 (21.2%)	88 (21.2%)	168 (24.4%)	23 (20.7%)	1257 (21.6%)
Missing	1327 (28.8%)	79 (19.0%)	57 (8.3%)	7 (6.3%)	1470 (25.2%)
**Age at Delivery (years)**					
Mean (SD)	29.9 (5.91)	32.4 (6.06)	30.7 (6.07)	32.7 (6.78)	30.2 (6.00)
Missing	< 5 (< 1%)	< 5 (< 1%)	< 5 (< 1%)	< 5 (< 1%)	< 10 (< 1%)
**Pre-pregnancy BMI Category**					
Overweight or obese	2135 (46.4%)	255 (61.3%)	284 (41.2%)	63 (56.8%)	2737 (47.0%)
Underweight or normal weight	2236 (48.5%)	120 (28.8%)	327 (47.5%)	32 (28.8%)	2715 (46.6%)
Missing	235 (5.1%)	41 (9.9%)	78 (11.3%)	16 (14.4%)	370 (6.4%)
**Delivery Mode**					
Cesarean	1230 (26.7%)	155 (37.3%)	184 (26.7%)	39 (35.1%)	1608 (27.6%)
Vaginal	3216 (69.8%)	249 (59.9%)	487 (70.7%)	71 (64.0%)	4023 (69.1%)
Missing	160 (3.5%)	12 (2.9%)	18 (2.6%)	1 (0.9%)	191 (3.3%)
**Preterm Birth (< 37 weeks’ gestation)**					
Preterm	295 (6.4%)	34 (8.2%)	64 (9.3%)	15 (13.5%)	408 (7.0%)
Term	4311 (93.6%)	382 (91.8%)	625 (90.7%)	96 (86.5%)	5414 (93.0%)

## Results

### Study population

The final sample consists of 5,822 participants; *N* = 527 diagnosed with GDM and *N* = 5,295 without GDM (Table 1). The prevalence of GDM in our analysis was 9.95%. Compared to the non-GDM group, a greater proportion of women with GDM self-identified as Asian (X^2^ = 26.73, *p* < 0.0001), mixed race (X^2^ = 46.50, *p* < 0.0001) or Hispanic (X^2^ = 122.13, *p* < 0.0005) and a lower proportion identified as Black (X^2^ = 34.74, *p* < 0.0001) or White (X^2^ = 10.28, *p* < 0.0005). The prevalence of GDM was 18.59% among women who self-identified as Asian, 17.14% among women who self-identified as Hispanic, and 15.76% among women who self-identified as mixed race. Women with GDM were more likely to report lower levels of educational attainment (X^2^ = 27.70, *p* < 0.0001) and were more likely to be overweight or obese prior to pregnancy compared to women without GDM (X^2^ = 79.34, *p* < 0.0001). Women with GDM were also more likely to deliver via cesarean (X^2^ = 24.78, *p* < 0.0001) or preterm (X^2^ = 4.28, *p* < 0.05). When dichotomized into non-clinical versus clinically elevated symptoms based on PROMIS-D cutoff scores, the prevalence of prenatal maternal depression in our analysis was 13.74%. When further grouping by *No GDM or Prenatal Maternal Depression (N* = 4,606), *GDM only* (*N* = 416), *Prenatal Maternal Depression Only* (*N* = 689), and *GDM and Prenatal Maternal Depression* (*N* = 111), sociodemographic differences persisted in race, ethnicity, educational attainment, pre-pregnancy weight, delivery mode, and in preterm delivery rates (Table 2).

### Association between GDM and prenatal maternal depression

Linear regression models showed that compared to women without GDM, women with GDM did not have significantly increased prenatal maternal PROMIS-D T-scores in unadjusted, partially adjusted, or fully adjusted models (*p*-values > 0.05). Based on dichotomized non-clinical (no prenatal depression) versus clinically elevated symptoms (prenatal depression) using PROMIS-D cutoff scores, a higher proportion of women with GDM (*N* = 527) were classified as having prenatal depression (*N* = 111; 21%) compared to the proportion of women without GDM who were classified as having prenatal depression (*N* = 689; 13%). However, logistic regression analyses to examine the likelihood of prenatal maternal depression among women with and without GDM were not significant (*p* > 0.05).

### Association between GDM, prenatal maternal depression, and postpartum depression

Linear regression models showed that women with *Prenatal Maternal Depression Only* and women with *Comorbid GDM and Prenatal Maternal Depression* had increased postpartum PROMIS-D T-scores in unadjusted (F(6, 5815) = 59.9, *p* < 0.0001, adj. *R*^2^ = 0.06), partially adjusted (F(23, 5798) = 19.28, *p* < 0.0001, adj. *R*^2^ = 0.07), and fully adjusted models (F(26, 5795) = 18.25, *p* < 0.0001, adj. *R*^2^ = 0.07) (Table 3). In the fully adjusted model, general linear hypothesis testing using Tukey pairwise contrasts showed women with *Prenatal Maternal Depression Only* (adjusted marginal mean 49.8, 95% CI 45.4, 54.2) had significantly increased postpartum PROMIS-D T-scores compared to women with *Neither GDM nor Prenatal Maternal Depression* (adjusted marginal mean 42.8, 95% CI 39.4, 48.1; mean difference 6.06, 95% CI 5.17, 6.94) (Table 3). Women with *Comorbid GDM and Prenatal Maternal Depression* (adjusted marginal mean 50.8, 95% CI 46.2, 55.4) also had significantly increased postpartum PROMIS-D T-scores compared to women with *Neither GDM nor Prenatal Maternal Depression* (mean difference 7.02, 95% CI 5.00, 9.05) (Table 3). However, there was no significant pairwise difference in postpartum PROMIS-D T-scores between women with *GDM only* as compared to *Neither GDM nor Prenatal Maternal Depression* (Table 3). Women with *Comorbid GDM and Prenatal Maternal Depression* also had significantly increased postpartum PROMIS-D T-scores compared to women with *GDM Only* (mean difference 6.71, 95% CI 4.51, 8.92), but not compared to *Prenatal Maternal Depression Only*.

**Table 3:** Association between GDM, prenatal maternal depression, and postpartum depression: pairwise contrasts

		Fully Adjusted^a^		
**Pairwise Comparisons**	**Mean Difference in Postpartum PROMIS T Scores ± Standard Error of Difference**	**Mean Difference (95% CI)**	***t-statistic***	***p-value***
*GDM Only* – *Neither GDM nor Prenatal Maternal Depression*	0.31 ± 0.42	-0.77, 1.40	0.73	0.47
*Prenatal Maternal Depression Only* – *Neither GDM nor Prenatal Maternal Depression*	6.06 ± 0.35	5.17, 6.94	17.29	**< 0.0001**
*GDM and Prenatal Maternal Depression* – *Neither GDM nor Prenatal Maternal Depression*	7.03 ± 0.80	5.00, 9.05	8.76	**< 0.0001**
*Prenatal Maternal Depression Only* – *GDM only*	5.74 ± 0.51	4.45, 7.04	11.23	**< 0.0001**
*GDM and Prenatal Maternal Depression – GDM Only*	6.71 ± 0.87	4.51, 8.92	7.70	**< 0.0001**
*GDM and Prenatal Maternal Depression* – *Prenatal Maternal Depression Only*	0.97 ± 0.84	-1.14, 3.08	1.16	0.25

^a^ Fully adjusted models include the original depression instrument, maternal race, ethnicity, age at delivery, educational attainment, gestational hypertension, pre-pregnancy body mass index, delivery mode, and preterm birth status

### GDM, Prenatal maternal depression, and odds of postpartum depression

**Fig. 2:**
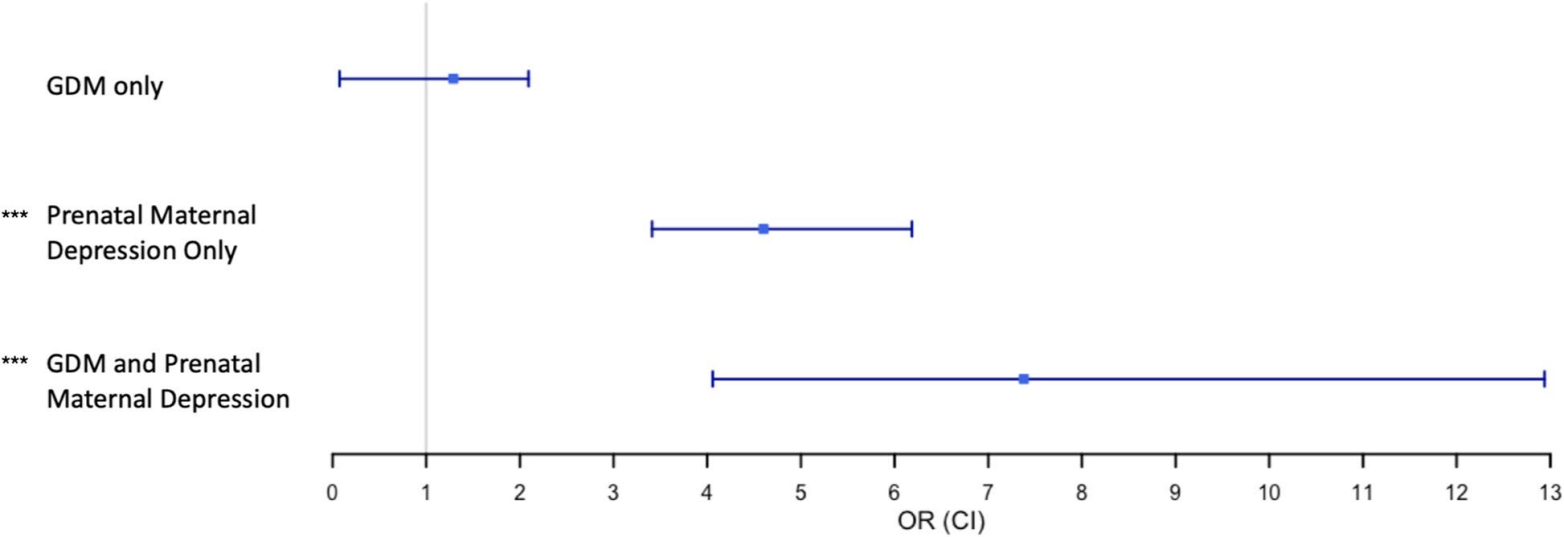
GDM, Prenatal Depression, and Likelihood of Postpartum Depression. The x-axis displays odds ratios (ORs) from fully adjusted models (controlling for the original depression instrument, maternal race, ethnicity, age at delivery, educational attainment, gestational hypertension, pre-pregnancy body mass index, delivery mode, and preterm birth status) and the 95% CI of the ORs for each GDM-depression category (y-axis). *** denotes significance at the 0.0001 level

Logistic regression was performed to ascertain the effects of GDM and prenatal maternal depression on postnatal maternal depressive symptoms when dichotomized into non-clinical (no PPD) versus clinically elevated symptoms (PPD) using PROMIS-D cutoff scores. Based on this categorization, the prevalence of PPD in our analysis was 5.55%. *Prenatal Maternal Depression Only* was associated with PPD in unadjusted (X^2^ = 119.6, *p* < 0.0001), partially adjusted (X^2^ = 102.9, *p* < 0.0001), and fully adjusted models (X^2^ = 101.2, *p* < 0.0001) (Table 4). *Comorbid GDM and Prenatal Maternal Depression* also was associated with PPD in unadjusted (X^2^ = 59.1, *p* < 0.0001), partially adjusted (X^2^ = 48.2, *p* < 0.0001), and fully adjusted models (X^2^ = 46.1, *p* < 0.0001) (Table 4). *GDM Only* was not associated with PPD in unadjusted, partially adjusted, or fully adjusted models (*p*-values > 0.05) (Table 4). In the fully adjusted model, *Comorbid GDM and Prenatal Maternal Depression* (OR 7.38, 95% CI 4.05, 12.94) was associated with an increased likelihood of PPD (Fig. 2). *Prenatal Maternal Depression Only* was also associated with an increased likelihood of PPD (OR 4.60, 95% CI 3.41, 6.18) (Fig. 2). However, *GDM Only* was not associated with an increased likelihood of PPD (OR 1.28, 95% CI 0.75, 2.09) (Fig. 2).

**Table 4:** Association between GDM, prenatal maternal depression, and likelihood of postpartum depression

			Fully Adjusted^a^				
**Groups**	**No PPD**	**PPD**	***z-statistic***	**X**^**2**^** Value**	***p-value***	**Odds Ratio**	**95% CI of Odds Ratio**
*Neither GDM nor Prenatal Maternal Depression*	4428	178	Reference	Reference	Reference	Reference	Reference
*GDM only*	398	18	0.98	0.96	0.32	1.28	0.75, 2.09
*Prenatal Maternal Depression Only*	598	91	10.06	101.2	**< 0.0001**	4.60	3.41, 6.18
*GDM and Prenatal Maternal Depression*	92	19	6.77	46.1	**< 0.0001**	7.38	4.05, 12.94
Overall	5516	306					

^a^ Fully adjusted models include the original depression instrument, maternal race, ethnicity, age at delivery, educational attainment, gestational hypertension, pre-pregnancy body mass index, delivery mode, and preterm birth status

## Discussion

To our knowledge the present analysis is the largest to date to examine associations among GDM and depression. It is also the first longitudinal analysis to examine the association between GDM and postpartum depression in the absence of prenatal maternal depression and, separately, comorbid GDM and prenatal maternal depression, with postpartum depression. In the present analysis, the prevalence of GDM and prenatal maternal depression were 9.95% and 13.74% respectively, which are comparable to the estimated GDM and prenatal maternal depression prevalence in the US [36, 37]. Similar to prior work [23], we observed participants with GDM were more likely to self-identify as Asian, mixed race, or Hispanic and were less likely to self-identify as White. In our analysis, the prevalence of GDM was 18.59% among women who self-identified as Asian, 17.14% among women who self-identified as Hispanic, and 15.76% among women who self-identified as mixed race, which reflect sociodemographic and geographic differences in the prevalence of GDM reported in the US [2] and globally [3]. However, the prevalence of PPD in our analysis was 5.55% which is lower than the estimated prevalence of PPD in the US [20]. This may be reflective of the EPDS to PROMIS-D harmonization underestimating depression or due to research referrals for perinatal depression treatment during the study resulting in a lower prevalence.

To summarize our findings examining the association between GDM and prenatal maternal depressive symptoms, in this analysis we did not find increased levels of prenatal maternal depressive symptoms in women with GDM compared to women without GDM. Based on dichotomized non-clinically relevant versus clinically relevant prenatal PROMIS-D T-scores, women with GDM had an increased prevalence of prenatal maternal depression using Chi Square analysis. However, in contrast to most prior analyses [21] our logistic regression analyses examining the likelihood of prenatal maternal depression among women with GDM was not significant after adjusting for covariates. There are several differences between our analysis and prior research to date that may account for the divergent findings. Some possible explanations include differences in prenatal maternal depression scores, differences in the timing of prenatal maternal depression measurement, and/or geographic or sociodemographic differences between cohorts.

In summary of our findings examining the joint and independent associations between GDM and prenatal maternal depression on postpartum maternal depressive symptoms and likelihood of PPD, as expected based on prior research, prenatal maternal depression was associated with increased postpartum depressive symptoms and an increased likelihood of PPD. As hypothesized, comorbid GDM and prenatal maternal depression was associated with increased postpartum maternal depressive symptoms compared to neither GDM nor prenatal maternal depression. However, in the absence of prenatal maternal depression, GDM was not associated with increased postpartum maternal depressive symptoms compared to neither GDM nor prenatal maternal depression. Finally, as hypothesized, we found that comorbid GDM and prenatal maternal depression was associated with a greater likelihood of PPD (OR 7.38, 95% CI 4.05, 12.94) compared to neither GDM nor prenatal maternal depression and the highest OR. However, GDM in the absence of prenatal maternal depression was not associated with an increased likelihood of PPD (OR 1.28, 95% CI 0.75, 2.09). The lack of association between GDM in the absence of prenatal maternal depression with postpartum maternal depressive symptoms and PPD suggests unmeasured prenatal maternal depression may have been a potential confound in prior analyses. Therefore, all three variables should be considered in subsequent analyses attempting to dissect these associations.

Significant strengths of our analysis include our large sample size leveraging the ECHO study that includes participant representation from thirteen US States and Puerto Rico, the diversity of our participant population with 43% of participants self-identifying as non-White and/or Hispanic, and having both a prenatal and postpartum assessment of maternal depressive symptoms. However, several limitations must be taken into consideration when interpreting our findings. There are several factors that should be explored in future analyses that we were unable to consider due constraints in harmonization across ECHO cohorts or the percentage of missing data. For example, we did not have information on the gestational week of GDM diagnosis, GDM treatment, the presence of GDM in a prior pregnancy, prior pregnancy losses, perinatal depression treatment such as therapies or pharmaceutical interventions, food insecurity, or social support during the perinatal period, which are all potential unobserved confounders. Data regarding parity, prenatal or postnatal mental health diagnoses, employment status, household income, and infant NICU status were missing for greater than 50% of participants. Additionally, there is some evidence of increased postpartum anxiety in women with GDM [38, 39], however harmonized anxiety measures were not available for participants included in the present analysis.

## Conclusions

Despite these limitations, our findings have important clinical and research implications: They underscore the importance of universal depression screening during pregnancy and through the first year postpartum [40]; identification of the interactions of different biological mechanisms underlying GDM, prenatal depression, and risk of PPD is needed to mitigate adverse maternal mental health outcomes. These findings suggest women with GDM and prenatal maternal depression should receive additional monitoring for postpartum mood disorders. Additionally, due to the joint association of GDM and prenatal maternal depression on risk of PPD, two conditions associated with increased subclinical levels of inflammation [6–10, 41, 42], future studies should examine potential mechanisms underlying this relation. Further understanding of mechanisms may inform prophylactic programs during pregnancy to prevent or treat postpartum mood disorders with the potential to improve long-term dyadic outcomes for mothers and their children.

## Supplementary Information

**Additional file 1.**

## Data Availability

The datasets for this manuscript are not publicly available because, per the NIH-approved ECHO Data Sharing Policy, ECHO-wide data have not yet been made available to the public for review/analysis. Requests to access the datasets should be directed to the ECHO Data Analysis Center, ECHO-DAC@rti.org.
