# Supplementary material for: Gestational diabetes mellitus, prenatal maternal depression, and risk for postpartum depression: an Environmental influences on Child Health Outcomes (ECHO) Study
Source: BMC Pregnancy Childbirth. 2022 Oct 8;22:758. doi: 10.1186/s12884-022-05049-4 (PMC9548153; doi:10.1186/s12884-022-05049-4)
Supplement: Supplementary file 1 — Additional file 1. [file 12884_2022_5049_MOESM1_ESM.docx]

**Supplementary Information**

**ECHO Cohort Data Collection Protocol Version 2.0:**

<https://dcricollab.dcri.duke.edu/sites/echomaterials/MOP%20and%20Protocol/ECHO-wide%20Cohort%20Data%20Collection%20Protocol%20Version2.0_24FEB2021_clean.pdf>

***Gestational Diabetes Mellitus (GDM).*** GDM status was ascertained via medical record abstraction for the majority of participants (*N* = 3096, 53.1%). A small portion of mothers self-reported on their GDM status (*N* = 325, 5.6%). Information on how GDM status was obtained was not available for the remaining N = 2401 participants (41.2%).

**ECHO Harmonization of Common Depression Assessments to the PROMIS Scale.** Data and information provided below are derived from the ECHO PRO Core Data Harmonization Group Harmonization Technical Report (3/26/2021 Version 4.2) ECHO PRO Core Data Harmonization Group (PROCoreDH@northwestern.edu). The PROMIS Depression T-scores are referenced to a mean of 50 and standard deviation of 10 with respect to the general adult US population, such that individuals with T-scores of 50 have depressive symptom severity equal to the mean in the general adult US population, individuals with T-scores of 60 have symptom severity 1 standard deviation above the mean. In the current analysis, data were originally collected using the EPDS (54%), PHQ-9 (25%), BSI (20%), and PROMIS (<1 %) for prenatal depression and using the EPDS (99%), CESD (<1 %), PHQ-9 (<1 %), and PROMIS (<1 %) for postnatal depression. Due to cohort differences in the timing of depression measurements across the prenatal and postnatal periods, participants with > 1 prenatal self-reported depression measurement and/or > 1 postnatal self-reported depression measurement during the first postpartum year had their prenatal and postnatal depression measurements averaged across the prenatal and postnatal period, respectively.

*Methods of Linking*. The following articles examined the psychometrics of harmonization between commonly used depression instruments to PROMIS-D (Blackwell et al., 2021; Choi, Schalet, Cook, & Cella, 2014; Kaat, Newcomb, Ryan, & Mustanski, 2017).

**Supplemental Results**

***Association between*** ***GDM, Prenatal Maternal Depression, and Postpartum Depression.*** Covariates significantly associated with postpartum PROMIS T-scores in the fully adjusted model included Hispanic ethnicity (mean difference -1.65, 95% CI -2.35, -0.94), pre-pregnancy BMI in the overweight or obese category (mean difference 0.46, 95% CI 0.008, 0.91), and preterm delivery (mean difference 1.30, 95% CI 0.48, 2.12). Post-hoc analyses demonstrated no interaction between Hispanic ethnicity, pre-pregnancy BMI in the overweight or obese category, or preterm delivery with Prenatal Maternal Depression Only, GDM only, or Comorbid Prenatal Maternal Depression and GDM on postpartum PROMIS T-scores (p-values > .05)

***Association between GDM, Prenatal Maternal Depression, and Postpartum Depression Stratified by Hispanic Ethnicity.*** Our primary analyses revealed women who self-identified as Hispanic had lower postpartum PROMIS-D T-scores compared to non-Hispanic women, therefore we conducted post-hoc analyses to examine the association between GDM, prenatal maternal depression, and postpartum depressive symptoms within a subset of participants who self-identified as Hispanic to determine if stratified results are consistent with our overall results. When stratifying by Hispanic ethnicity, linear regression models revealed that compared *Neither GDM nor Prenatal Maternal Depression (N* = 768), women with *Prenatal Maternal Depression Only (N = 247)* and women with *Comorbid GDM and Prenatal Maternal Depression (N* = 58) had increased postpartum PROMIS-D T-scores in fully adjusted models (F(18, 1206) = 8.47, *p* < .0001, adj. R^2^ = 0.09) in women who self-identify as Hispanic. In the fully adjusted model, general linear hypothesis testing using Tukey pairwise contrasts revealed women with *Prenatal Maternal Depression Only* (adjusted marginal mean 51.3, 95% CI 41.8, 54.5) had significantly increased postpartum PROMIS-D T-scores compared to women with *Neither GDM nor Prenatal Maternal Depression* (adjusted marginal mean 44.7, 95% CI 41.7, 47.8; mean difference 6.55, 95% CI 4.92, 8.19). Women with *Comorbid* *GDM and Prenatal Maternal Depression* (adjusted marginal mean 51.5, 95% CI 47.7, 55.3) also had significantly increased postpartum PROMIS-D T-scores compared to women with *Neither GDM nor Prenatal Maternal Depression* (mean difference 6.74, 95% CI 3.72, 9.77). There was no significant pairwise difference in postpartum PROMIS-D T-scores between women with *GDM only* (*N* = 152, adjusted marginal mean 45.0, 95% CI 41.7, 48.3) as compared to *Neither GDM nor Prenatal Maternal Depression.* Women with *Comorbid GDM and Prenatal Maternal Depression* also had significantly increased postpartum PROMIS-D T-scores compared to women with *GDM Only* (mean difference 6.70, 95% CI 3.38, 10.03), but not compared to *Prenatal Maternal Depression Only*. Covariates significantly associated with postpartum PROMIS T-scores in the fully adjusted model included and preterm birth (mean difference 2.25, 95% CI 0.46 – 4.04).

***Association between GDM, Prenatal Maternal Depression, and Postpartum Depression Stratified by Overweight or Obese BMI.*** Our primary analyses revealed women with pre-pregnancy BMI’s categorized as overweight or obese had higher PROMIS-D T-scores compared to women with pre-pregnancy BMI’s categorized within the healthy weight range. We therefore conducted post-hoc analyses to examine the association between GDM, prenatal maternal depression, and postpartum depressive symptoms within a subset of participants with pre-pregnancy BMI’s categorized as overweight or obese to determine if stratified results are consistent with our overall results. When stratifying by overweight or obese pre-pregnancy BMI, linear regression models revealed that compared *Neither GDM nor Prenatal Maternal Depression (N* = 2135), women with *Prenatal Maternal Depression Only (N = 284)* and women with *Comorbid GDM and Prenatal Maternal Depression (N* = 63) had increased postpartum PROMIS-D T-scores in the fully adjusted model (F(11, 2725) = 19.26, *p* < .0001, adj. R^2^ = 0.07). In the fully adjusted model, general linear hypothesis testing using Tukey pairwise contrasts revealed women with *Prenatal Maternal Depression Only* (adjusted marginal mean 50.6, 95% CI 48.9, 52.2) had significantly increased postpartum PROMIS-D T-scores compared to women with *Neither GDM nor Prenatal Maternal Depression* (adjusted marginal mean 44.4, 95% CI 42.0, 45.8; mean difference 6.20, 95% CI 4.85, 7.54). Women with *Comorbid* *GDM and Prenatal Maternal Depression* (adjusted marginal mean 52.5, 95% CI 50.0, 55.0) also had significantly increased postpartum PROMIS-D T-scores compared to women with *Neither GDM nor Prenatal Maternal Depression* (mean difference 8.11, 95% CI 5.37, 10.85). There was no significant pairwise difference in postpartum PROMIS-D T-scores between women with *GDM only* (*N* = 255, adjusted marginal mean 45.2, 95% CI 43.5, 46.9) as compared to *Neither GDM nor Prenatal Maternal Depression.* Women with *Comorbid GDM and Prenatal Maternal Depression* also had significantly increased postpartum PROMIS-D T-scores compared to women with *GDM Only* (mean difference 7.31, 95% CI 4.34, 10.28), but not compared to *Prenatal Maternal Depression Only*.

***GDM, Prenatal Maternal Depression, and Odds of Postpartum Depression*.** Other significant predictors of PPD in the fully adjusted model included pre-pregnancy BMI categorized as being overweight or obese (OR 1.37, 95% CI 1.05, 1.78) and preterm delivery (OR 1.72, 95% CI 1.16, 2.47).

References:

Blackwell, C. K., Tang, X., Elliott, A. J., Thomes, T., Louwagie, H., Gershon, R., . . . Cella, D. (2021). Developing a common metric for depression across adulthood: Linking PROMIS depression with the Edinburgh Postnatal Depression Scale. *Psychol Assess, 33*(7), 610-618. doi:10.1037/pas0001009

Choi, S. W., Schalet, B., Cook, K. F., & Cella, D. (2014). Establishing a common metric for depressive symptoms: linking the BDI-II, CES-D, and PHQ-9 to PROMIS depression. *Psychol Assess, 26*(2), 513-527. doi:10.1037/a0035768

Kaat, A. J., Newcomb, M. E., Ryan, D. T., & Mustanski, B. (2017). Expanding a common metric for depression reporting: linking two scales to PROMIS((R)) depression. *Qual Life Res, 26*(5), 1119-1128. doi:10.1007/s11136-016-1450-z
